# Association between insecticide exposomics and cognitive function in older adults: an observational study based on NHANES 2011–2014

**DOI:** 10.3389/fpubh.2025.1556263

**Published:** 2025-07-09

**Authors:** Mengfan Zhang, Xiaofang Guo, Yuanjun Chen, Xinghua Liu, Xingdong Lin

**Affiliations:** ^1^Guangzhou University of Traditional Chinese Medicine, Guangzhou, China; ^2^The Third Affiliated Hospital of Guangzhou University of Traditional Chinese Medicine, Guangzhou, China

**Keywords:** insecticide exposomics, cognitive function, older adult population, NHANES data, pesticide exposure, cognitive decline, public health research

## Abstract

**Objective:**

This study aims to investigate the association between exposure to insecticide exposomics and cognitive function, among adults aged 60 and above.

**Methods:**

We employed a multi-stage probability sampling method to analyze data derived from the National Health and Nutrition Examination Survey (NHANES) conducted between 2011 and 2014. The study investigated the impact of insecticides exposure on cognitive performance, as measured by the Consortium to Establish a Registry for Alzheimer’s Disease (CERAD) Word Learning Test (CERAD-WL), CERAD Delayed Recall Test (CERAD-DR), Animal Fluency Test (ATF), and Digit Symbol Substitution Test (DSST). Logistic regression models were used to evaluate the impact, with adjustments for sociodemographic and lifestyle variables, and subgroup analyses were conducted to further elucidate the findings.

**Results:**

A total of 1,544 participants were included after applying strict exclusion criteria. Logistic regression analysis disclosed a significant association between insecticides exposure and the risk of cognitive impairment. Specifically, for CERAD-WL, the unadjusted model showed an Odds Ratio (OR) of 0.68 (95% CI: 0.49–0.93), *p* = 0.0174, and the adjusted model showed an OR of 0.71 (95% CI: 0.51–0.99), *p* = 0.0466. CERAD-DR demonstrated an unadjusted OR of 0.64 (95% CI: 0.46–0.88), *p* = 0.0060, and an adjusted OR of 0.67 (95% CI: 0.47–0.94), *p* = 0.021. The DSST indicated a correlation with pesticide exposure in the unadjusted model with *p* = 0.0214. Herbicides were notably associated with ATF in the unadjusted model with an OR of 1.70 (95% CI: 1.14–2.53), *p* = 0.0093. However, after adjusting for sociodemographic and lifestyle variables, some associations were no longer statistically significant. For instance, the association between insecticides exposure and CERAD-WL performance became non-significant (OR: 0.80, 95% CI: 0.56–1.15, *p* = 0.2284), and similarly for CERAD-DR (OR: 0.72, 95% CI: 0.50–1.03, *p* = 0.0734).

**Conclusion:**

Our study indicates an association between insecticides exposure and cognitive impairment, particularly affecting memory and delayed recall. Yet, the cross-sectional design prevents conclusive causality claims. Future research should adopt longitudinal approaches, utilize biological markers for precise exposure measurement, and apply advanced statistical techniques to better understand the exposure-outcome relationship. Including a broader age range and detailed confounder data will also strengthen causal inferences. While our findings offer preliminary evidence, more robust studies are necessary to confirm causality and guide preventive measures.

## 1 Introduction

Cognitive decline, predominantly affecting individuals aged 60 and above, is characterized by a diminished capacity for daily activities and a significant reduction in social functioning. It is considered an intermediate stage between normal aging and dementia (1–3). As one age, cognitive decline often progresses to dementia, primarily in the form of Alzheimer’s disease (AD), with an estimated annual rate ranging from 5 to 17% (4). AD affects an estimated 50 million people worldwide, a number expected to triple by 2050, driven largely by the aging global population (5). Currently, the annual global cost of dementia exceeds $1.3 trillion and is projected to rise to $2.8 trillion by 2030 (6). However, effective treatments for dementia remain elusive, and early-stage diagnosis lacks definitive indicators. Focusing on cognitive function is a novel target, as some causes of cognitive decline might be reversible or potentially treatable. Therefore, leveraging cognitive test performance in the older adult to predict cognitive function levels and identify risk factors for cognitive decline offers a potential avenue for early intervention (1, 7).

Pesticides, defined as substances or mixtures of substances used for the prevention, destruction, removal, mitigation of any pest, include insecticides, herbicides, fungicides and many other types. These pesticides contain a variety of chemical structures and are commonly used in household and outdoor products (8, 9). However, their use can lead to food and water contamination, increasing the risk of exposure to these substances and potentially causing harm to human health (10, 11). Pesticide exposure has been linked to multiple diseases, including Hodgkin’s disease (HD), non-Hodgkin’s lymphoma (NHL), and Parkinson’s disease, as well as disorders affecting the endocrine, respiratory, and reproductive systems (12, 13).

Mechanistically, insecticides inhibit acetylcholinesterase (AChE), causing synaptic acetylcholine accumulation and neuronal overexcitation (14). In chronic exposure scenarios, this triggers secondary cascades involving oxidative stress, neuroinflammation, and mitochondrial dysfunction, ultimately leading to synaptic loss and neuronal apoptosis (15, 16). Animal models corroborate these pathways: Malathion exposure induces hippocampal oxidative stress and spatial memory deficits in rats (17), while rotenone disrupts blood–brain barrier integrity, activates microglial, and triggering neuronal apoptosis in mice (18). Crucially, even subacute paraquat administration promotes complement-mediated synaptic pruning, highlighting the diversity of pesticide-induced neurotoxic mechanisms (19).

Human studies further support this association. Event-related potential (ERP) analyses reveal persistent neurophysiological deficits in acute insecicides poisoning survivors, including delayed attentional processing lasting up to 6 months post-exposure (20). Chronic low-dose effects are suggested by NHANES-based analyses, where elevated urinary 3-phenoxybenzoic acid (3-PBA), a pyrethroid metabolite, correlates with poorer cognitive scores in older adults (21). However, population-level evidence specifically linking insecticides to cognitive decline remains scarce, particularly regarding daily low-dose exposure in aging populations.

To further understand the effects of pesticides on cognitive function, this study utilizes data from the National Health and Nutrition Examination Survey (NHANES) in the United States to assess the relationship between the use of insecticides and cognitive function levels in older adults, aiming to provide key epidemiological insights. The NHANES is a series of investigations designed to assess the health and nutritional status of the U.S. population. Utilizing a layered, probabilistic sampling method, it pinpoints a sample that mirrors the national demographic of the civilian, noninstitutionalized population in the U.S.

## 2 Methods

### 2.1 Study design and ethical considerations

Our research primarily uses data from the NHANES database. NHANES provides a comprehensive overview of the socioeconomic, health, behavioral, and nutritional aspects of both adults and children within the U.S. populace. Participants undergo a comprehensive examination at a mobile examination center (MEC) after a household interview, which includes a physical examination, specialized measurements, and laboratory tests. Acknowledged for its reliability and multifaceted nature, this approach enables the creation of nationwide estimates. Further information regarding the NHANES database and the methodology of data collection can be accessed at the official NHANES website.^1^

### 2.2 Pesticide use

To obtain an accurate assessment of insecticides usage, we collected data from NHANES on all pesticides used by individuals in domestic, horticultural, and garden contexts over the past 7 days. One of the questions posed during the interview was: “Has any chemical product been used in your home to control fleas, cockroaches, ants, termites, or other insects within the past 7 days?” Additionally, participants were asked: “In the past 7 days, were any chemical products used in your lawn or garden to kill weeds?” While self-reported data provide a useful proxy for exposure, they are subject to misclassification bias.

Participants’ responses in 2011–2012 can be categorized as follows: A total of 716 respondents indicated that they had used a chemical product in their home to control insects, while 6,439 respondents stated that they had not. Additionally, 666 respondents indicated that they did not know or provided an invalid response. Similarly, in 2013–2014, 789 respondents reported using a chemical product to control fleas, roaches, ants, termites, or other insects within the past 7 days. However, 6,991 respondents stated that they had not used such a product, while 511 respondents indicated that they did not know or provided an invalid response.

The other question was “In the past 7 days, were any chemical products used in your lawn or garden to kill weeds?” A total of 260 respondents indicated that they had used a chemical product in their lawn or garden to kill weeds, while 6,818 respondents stated that they had not used such a product in 2011–2012. Additionally, in 2013–2014, 424 respondents indicated that they had used such products, while 7,258 respondents indicated that they had not used a chemical product in their lawn or garden to kill weeds.

### 2.3 Cognitive assessment

Assessing cognitive function involves either home interviews or comprehensive evaluations at mobile examination centers. Tools like the CERAD Word Learning Test (CERAD-WL), CERAD Delayed Recall Test (CERAD-DR), Animal Fluency Test (ATF), and Digit Symbol Substitution Test (DSST) are employed to provide a comprehensive evaluation of cognitive abilities.

The CERAD assesses memory capability by testing the retention of new verbal information over time, consisting of three consecutive immediate learning trials with delayed recall tests. During the CERAD learning phases, participants are required to verbalize ten distinct words (22, 23). After the Animal Fluency test (AFT) and DSST assessments, participants proceed to the delayed word recall segment. Each trial is awarded a score on a scale of 10, and the composite CERAD score is the total of all trial scores.

Concurrently, the AFT assesses executive function by evaluating categorical verbal fluency. Participants are tasked with enumerating as many animals as possible within 1 min, with scores typically ranging from 3 to 39 (24). The DSST, part of the Wechsler Adult Intelligence Scale, is designed to assess processing speed, attention, and working memory. The assessment employs a paper-based format comprising a key with nine digits, each associated with a distinct symbol. Participants are permitted a two-minute timeframe in which to accurately transfer the corresponding symbols into the 133 allocated boxes adjacent to the numbers. The test is scored based on the number of correct pairings, with a maximum achievable score of 105 (25).

Scoring criteria for cognitive impairment are typically set at 25% below the total possible score. Consistent with this standard, potential cognitive impairment is indicated by scores below 17 for CERAD-WL, below 5 for CERAD-DR, below 14 for ATF, and below 34 for DSST (26, 27). This threshold aligns with prior NHANES-based research, which has utilized similar cutoffs to identify cognitive impairment in older adult populations (28). Adopting these established thresholds enables a standardized approach to classifying cognitive impairment, facilitating comparisons across studies, and enhancing the generalizability of our findings.

### 2.4 Covariates

This study evaluated various potential confounders, including sociodemographics (age, gender, race/ethnicity, education, marital status, income, and Poverty Income Ratio [PIR]), lifestyle factors (smoking, alcohol, BMI, and sleep), and medical conditions (hypertension, diabetes, heart failure, stroke, and depression).

Participants were categorized by age groups (60–69, 70–79, and 80+), gender (male/female), and race and ethnicity (Mexican American, other Hispanic, non-Hispanic Caucasian, non-Hispanic African American, and others). Education levels were divided into those without a high school diploma and those with higher education. Marital status was categorized as never married, married/cohabiting, widowed, divorced, or separated. Household income was split into four brackets: under $20,000, $20,000–$45,000, $45,000–$75,000, and over $75,000. PIR was categorized as low (below 5) or high (5 or above).

Lifestyle factors were assessed as follows: smokers were defined as those with at least 100-lifetime cigarettes; alcohol use was based on at least 12 annual drinks; BMI was classified as normal (under 25 kg/m^2^), overweight (25–30 kg/m^2^), or obese (30 kg/m^2^ or more); and sleep duration was self-reported and categorized as 10 h or less, or over 10 h.

Medical conditions were identified through self-reported diagnoses by healthcare providers for hypertension, diabetes, heart failure, and stroke, and depressive symptoms were assessed using the PHQ-9, with scores of 10 or higher indicating depression.

To assess the robustness of our findings, we conducted sensitivity analyses to exclude participants with missing data. In the analysed tables it was shown that the ORs of insecticides with the CERAD-DR and DSST tests remained statistically significant (*p*-values of 0.014 and <0.001, respectively).

Missing data rates were low across variables: alcohol (0.39%), BMI (1.42%), education (0.06%), marital status (0.06%), income (4.53%), PIR (7.57%), diabetes (0.06%), heart disease (0.26%), stroke (0.19%), hypertension (0.26%), sleep duration (0.25%), and smoking status (0.06%). Multiple imputation was performed using the “MICE” package in R, following the methodology of Van Buuren and Groothuis-Oudshoorn.

### 2.5 Statistical analysis

Our statistical approach encompassed sample weights, clustering, and stratification, which are crucial for estimating national trends and enhancing the generalizability of our findings. To assess the normality of the data, we employed the Kolmogorov–Smirnov test. Categorical variables were reported as counts and percentages, while normally distributed continuous variables were presented as mean ± standard error (SE), and non-normally distributed continuous variables were described using the median and interquartile range (IQR). For comparative tests, we used the Student’s t-test for the mean values of normally distributed continuous variables between groups, the Mann–Whitney U test for non-normally distributed continuous variables, and chi-square tests for categorical variable comparisons.

Furthermore, we conducted a logistic regression analysis to explore the relationship between pesticide exposure and cognitive function, which included an unadjusted model (Model 1), a model adjusted for basic demographic variables (Model 2), and a model that encompassed a broader range of factors (Model 3). ORs and their corresponding 95% CIs were determined. To further investigate potential interactions, we performed subgroup analyses using eXtreme Statistical Software 4.1, assessing the interactive effects of covariates such as age, gender, income, alcohol consumption, BMI, and hypertension on cognitive function. We also used the Research Analytics website for sensitivity analyses, and GraphPad Prism 10.1.2 to make clearer dot plots of *p*-values in the research demographic characteristics.

## 3 Results

### 3.1 Study population

**Figure 1:**
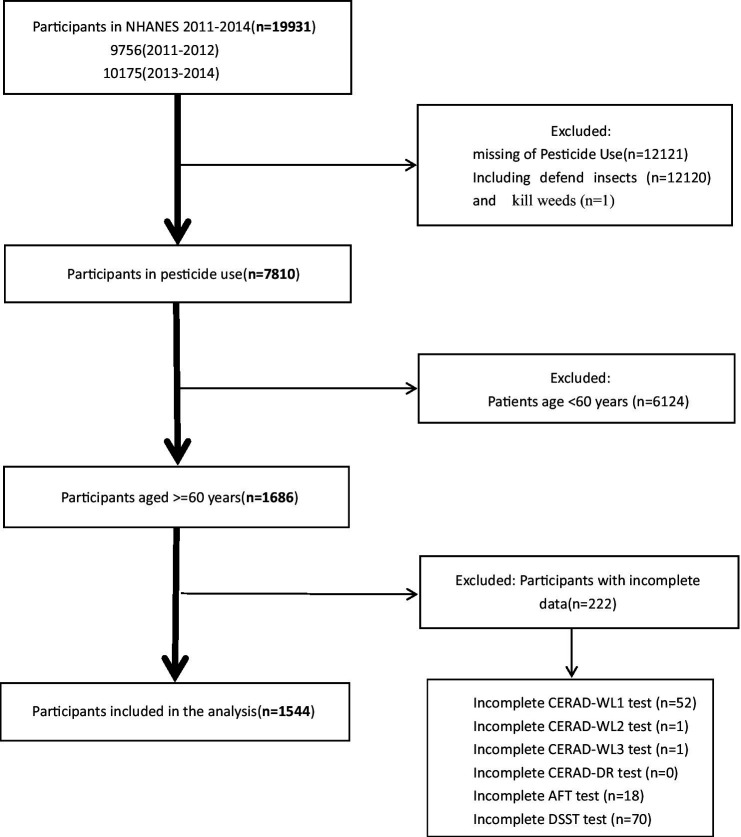
Flowchart of the sample selection from the National Health and Nutrition Examination Survey (NHANES).

A comprehensive sample of 19,931 individuals was initially included from the 2011–2012 and 2013–2014 cycles of the NHANES. Subsequently, participants with incomplete data regarding pesticide exposure were systematically excluded: totaling 12,121 individuals (12,120 defends insects and 1 kills weeds). Following this, those lacking results from four key dementia assessment tests were excluded: 142 individuals (54 CERAD-WL test; 0 CERAD-DR test; 18 AFT test and 70 DSST test). Additionally, individuals under the age of 60 were excluded, totaling 6,124 individuals. This strict exclusion process resulted in a high-quality dataset comprising 1,544 participants (Figure 1). Since this article focuses on mainly insecticides and the sample size regarding herbicide use after screening, the sample was too small to be convincing, so the discussion focuses on the relationship between insecticides and cognitive function.

### 3.2 Demographic characteristics of the study population

Table 1 presents the demographics and characteristics data of the 1,544 participants, categorized based on CERAD-WL scores and insecticides exposure. Among these participants, 217 were reported to have used insecticides pesticide in the past week. With a CERAD-WL score of 17 as the cognitive decline threshold, the age distribution was as follows: 53.4% were aged 60–70 years, 29.8% were aged 70–80 years, and 16.8% were over 80 years old. The cohort was 52% female and 48% male, with the majority of participants (51.2%) being non-Hispanic white, non-Hispanic black (20.4%), Mexican American (12.4%), and other races (16%). Ninety point 5 % (90.5%) had at least a high school diploma. Regarding marital status, 5.4% were unmarried, 58.8% were married or cohabiting, and 35.8% were widowed, divorced, or separated.

**Table 1:** Demographic characteristics of participants in the National Health and Nutrition Examination Survey conducted in the US of the 2011–2012 and 2013–2014 cycles.

Characteristics (CERAD-WL)	Has used Pesticide (*N* = 217)		*p*- Value	Lacks used pesticide (*N* = 1,327)		*p*- value
	> = 17	<17		> = 17	<17	
Age (years) Age categories			**0.095**			**<0.001**
60–70	93 (60.39%)	28 (44.44%)		595 (57.27%)	109 (37.85%)	
70–80	40 (25.97%)	24 (38.10%)		304 (29.26%)	93 (32.29%)	
>80	21 (13.64%)	11 (17.46%)		140 (13.47%)	86 (29.86%)	
Gender *n* (%)			**0.006**			**<0.001**
Male	64 (41.56%)	39 (61.90%)		470 (45.24%)	169 (58.68%)	
Female	90 (58.44%)	24 (38.10%)		569 (54.76%)	119 (41.32%)	
Race			**0.232**			**0.054**
Mexican American	26 (16.88%)	7 (11.11%)		119 (41.32%)	39 (13.54%)	
Other Hispanic	23 (14.94%)	9 (14.29%)		74 (7.12%)	29 (10.07%)	
Non-Hispanic White	64 (41.56%)	21 (33.33%)		564 (54.28%)	141 (48.96%)	
Non-Hispanic Black	36 (23.38%)	21 (33.33%)		204 (19.63%)	54 (18.75%)	
Other Race	5 (3.25%)	5 (7.94%)		105 (10.11%)	25 (8.68%)	
Education level			**0.021**			**<0.001**
Less than high school	18 (11.69%)	17 (26.98%)		60 (5.77%)	50 (17.36%)	
High school	69 (44.81%)	23 (36.51%)		369 (35.51%)	126 (43.75%)	
More than high school	67 (43.51%)	23 (36.51%)		609 (58.61%)	112 (38.89%)	
Married			**0.129**			**0.538**
Never married	12 (7.79%)	3 (4.76%)		52 (5.00%)	16 (5.56%)	
Married or living with a partner	84 (54.55%)	27 (42.86%)		634 (61.02%)	163 (56.60%)	
Widowed or divorced or separated	58 (37.66%)	33 (52.38%)		352 (33.88%)	109 (37.85%)	
Annual family income ($)			**0.066**			**<0.001**
<$20,000	41 (26.62%)	23 (36.51%)		200 (19.25%)	84 (29.17%)	
$20,000 to <$45,000	51 (33.12%)	17 (26.98%)		338 (32.53%)	100 (34.72%)	
$45,000 to <$75,000	28 (18.18%)	8 (12.70%)		200 (19.25%)	43 (14.93%)	
≥$75,000	29 (18.83%)	8 (12.70%)		258 (24.83%)	46 (15.97%)	
Missing	5 (3.25%)	7 (11.11%)		43 (4.14%)	15 (5.21%)	
Poverty income ratio (%)			**0.163**			**0.001**
<5	131 (85.06%)	58 (92.06%)		826 (79.50%)	253 (87.85%)	
> = 5	23 (14.94%)	5 (7.94%)		213 (20.50%)	35 (12.15%)	
Smoking status			**0.977**			**0.731**
Yes	81 (52.60%)	33 (52.38%)		525 (50.53%)	140 (48.61%)	
No	73 (47.40%)	30 (47.62%)		513 (49.37%)	148 (51.39%)	
Alcohol consumption			**0.312**			**0.102**
Yes	100 (64.94%)	42 (66.67%)		722 (69.49%)	187 (64.93%)	
No	53 (34.42%)	19 (30.16%)		314 (30.22%)	98 (34.03%)	
Missing	1 (0.65%)	2 (3.17%)		3 (0.29%)	3 (1.04%)	
Body mass weight (kg/m^2^)			**0.883**			**0.018**
Normal weight (<25)	38 (24.68%)	17 (26.98%)		272 (26.18%)	78 (27.08%)	
Overweight (25 to <30)	62 (40.26%)	26 (41.27%)		365 (35.13%)	123 (42.71%)	
Obese (> = 30)	54 (35.06%)	20 (31.75%)		402 (38.69%)	87 (30.21%)	
Sleep Duration (hours)			**0.265**			**0.598**
<=10 (hours)	151 (98.05%)	63 (100.00%)		1,038 (99.90%)	288 (100.00%)	
>10 (hours)	3 (1.95%)	0 (0.00%)		1 (0.10%)	0 (0.00%)	
Hypertention			**0.806**			**0.318**
Yes	111 (72.08%)	45 (71.43%)		641 (61.69%)	188 (65.28%)	
No	42 (27.27%)	17 (26.98%)		397 (38.21%)	99 (34.38%)	
Diabetes history			**0.679**			**0.070**
Yes	40 (25.97%)	19 (30.16%)		286 (27.53%)	95 (32.99%)	
No	113 (73.38%)	44 (69.84%)		753 (72.47%)	193 (67.01%)	
Missing	1 (0.65%)	0 (0.00%)				
Heart failure			**0.506**			**0.012**
Yes	14 (9.09%)	4 (6.35%)		60 (5.77%)	31 (10.76%)	
No	140 (90.91%)	59 (93.65%)		976 (93.94%)	256 (88.89%)	
Stroke			**0.030**			**0.081**
Yes	10 (6.49%)	10 (15.87%)		60 (5.77%)	27 (9.38%)	
No	144 (93.51%)	53 (84.13%)		977 (94.03%)	260 (90.28%)	
Depression score			**0.314**			**0.010**
No depression	21 (13.64%)	12 (19.05%)		94 (9.05%)	41 (14.24%)	
Had depression	133 (86.36%)	51 (80.95%)		945 (90.95%)	247 (85.76%)	

Sum of weights = 1,544. The bold black font indicates the *p*-value.

Economically, 72.9% of the participants had incomes between $20,000 and $75,000, 22.5% earned under $20,000, and 82.1% lived below the poverty line. Lifestyle factors showed 50.4% as smokers and 68% as alcohol consumers. In terms of BMI categories, 26.2% were normal, 37.4% were overweight, and 36.4% were obese, with 99.7% reporting sleeping 10 h or less per day. Medically, 63.8% had hypertension, 28.5% had diabetes, 7% had heart disease, and 6.9% had a stroke. PHQ-9 scores indicated 89.1% with depressive symptoms (score ≥10).

**Figure 2:**
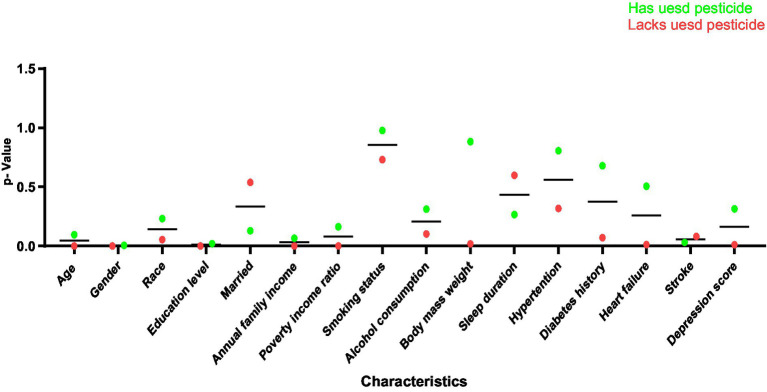
*p*-value dot plots of demographic characteristics of participants in the National Health and Nutrition Examination Survey conducted in the US of the 2011–2012 and 2013–2014. Sum of weights = 1,544.

Figure 2 is a dot plot to show the statistical significance (*p*-value) between different characteristics (Characteristics) and whether pesticides are used or not (Has used pesticide/Lacks used pesticide), which makes it easy to see the correlation between the data more clearly.

### 3.3 The association between insecticides exposure and increased cognitive function risk

Logistic regression analysis revealed significant associations between insecticides exposure and cognitive impairment in the unadjusted models. Pesticide use is divided into insecticides and herbicides. Model 1 was unadjusted, Model 2 adjusted for basic demographics, and Model 3 included a broader range of factors such as education, income, health behaviors, and medical history. However, after adjusting for sociodemographic and lifestyle variables, some of these associations were no longer statistically significant. The association was significant for CERAD-WL (unadjusted: 0.68, *p* = 0.0174; adjusted: 0.71, *p* = 0.0466) and CERAD-DR (unadjusted: 0.64, *p* = 0.0060; adjusted: 0.67, *p* = 0.021). DSST also showed a correlation with pesticide exposure (unadjusted: *p* = 0.0214). Herbicides had a notable association with ATF (unadjusted: 1.70, *p* = 0.0093; adjusted: 1.32, *p* = 0.2181). These findings suggest that there may be some association between insecticides exposure and increased risk of cognitive functioning (Table 2), but it is worth noting that the study only differentiated between pesticide use and has not yet considered in depth the differences in their chemical structure. So we analysed three of the more common urinary metabolites based on the data available inside the Nhanes database. As for pyrethroid insecticides and organophosphorus insecticides, which are the two most common types of insecticides, I chose the more representative ones, para-nitrophenol (PNP) and Trans-3-(2,2-dichlorovinyl)-2,2-dimethylcyclopropane carboxylic acid (Trans-DCCA). For the sake of comprehensiveness of the article’s findings, I also examined a herbicide, 2,4-dichlorophenoxyacetic acid (2,4-D) (Supplementary Tables 2–4). In addition, we did sensitivity analyses showing that even without some missing data, these results remain statistically significant and are unlikely to lose significance altogether due to missing data (Supplementary Table 1). Due to space limitations and the focus of this paper, the analysis of herbicide exposure is only presented in Table 2. The other tables in this study focus solely on the analysis of insecticide exposure. This selective presentation does not imply lesser importance of herbicides but rather a focused approach given the constraints of the study’s scope and available data.

**Table 2:** Multivariate logistic regression between include insecticides exposure and increased cognitive function risk in the National Health and Nutrition Examination Survey conducted in the US between 2011 and 2014.

	Model 1 OR (95%), *p*	Model 2 OR (95%), *p*	Model 3 OR (95%), *p*
CERAD-WL(<17)			
Defend insect			
Yes	1	1	1
No	0.68 (0.49, 0.93) **0.017**	0.71 (0.51, 0.99) **0.046**	0.80 (0.56, 1.15) **0.228**
Kill weeds			
Yes	1	1	1
No	1.01 (0.69, 1.50) **0.940**	1.14 (0.76, 1.72) **0.534**	0.91 (0.59, 1.40) **0.676**
CERAD-DR(<5)			
Defend insect			
Yes	1	1	1
No	0.64 (0.46, 0.88) **0.006**	0.67 (0.47, 0.94) **0.021**	0.72 (0.50, 1.03) **0.073**
Kill weeds			
Yes	1	1	1
No	1.15 (0.76, 1.74) **0.502**	1.35 (0.87, 2.09) **0.183**	1.10 (0.69, 1.74) **0.686**
ATF(<14)			
Defend insect			
Yes	1	1	1
No	0.80 (0.59, 1.09) **0.160**	0.89 (0.64, 1.23) **0.475**	1.10 (0.77, 1.56) **0.605**
Kill weeds			
Yes	1	1	1
No	1.70 (1.14, 2.53) **0.009**	1.62 (1.06, 2.46) **0.024**	1.32 (0.85, 2.06) **0.212**
DSST(<34)			
Defend insect			
Yes	1	1	1
No	0.69 (0.50, 0.94) **0.018**	0.87 (0.61, 1.22) **0.408**	1.26 (0.83, 1.91) **0.270**
Kill weeds			
Yes	1	1	1
No	1.33 (0.89, 2.00) **0.164**	1.32 (0.85, 2.05) **0.218**	0.82 (0.50, 1.33) **0.411**

Correlation coefficients of *β* (95%CI) *p*-value / OR (95%CI) *p*-value are used in the table Non-adjusted model adjust for: None; Adjust I model adjust for: AGE; GENDER; RACE; Adjust II model adjust for: ALCOHOL; BMI; GENDER; RACE; AGE; EDUCATION; MARRIED; ANNUAL.FAMILY.INCOME; POVERTY.INCOME.RATIO; PHQ; DMHISTORY; GENERAL.HEALTH. CONDITION; HEART.FAILURE; STROKE; HYPERTENSION; VIGOROUS.WORK.ACTIVITY; MODERATE. WORK.ACTIVITY; SLEEP.HOUR; SMOKING. The bold black font indicates the *p*-value.

### 3.4 Associations between insecticides exposure and increased cognitive function risk and vulnerable subgroups

Subgroup analyses using eXtreme Statistical Software (XSS) investigated the interactions between cognitive function and various covariates, including age, gender, income, alcohol, BMI, and hypertension. The covariates were categorized according to the criteria outlined in Table 1. P-interaction values were used to assess the significance of these interactions on cognitive outcomes. For DSST, p-interactions were: age (0.785), gender (0.338), income (0.688), alcohol (0.925), BMI (0.125), and hypertension (0.453). For CERAD-WL, the corresponding values were: age (0.369), gender (0.398), income (0.298), alcohol (0.584), BMI (0.992), and hypertension (0.600). For AFT, p-interaction values were: age (0.518), gender (0.649), income (0.513), alcohol (0.872), BMI (0.253), and hypertension (0.986). The results indicate that the majority of covariates exhibit minimal interactive effects on cognitive outcomes, suggesting that they may not significantly influence cognitive performance in this study (Table 3).

**Table 3:** Subgroup analyses of the association between insecticides exposure and increased cognitive function risk in the National Health and Nutrition Examination Survey conducted in the US between 2011 and 2014.

	DSST test (<34)		CERAD-WL (<17)		CERAD-DR (<5)		AFT (<14)	
	OR (95% CI)	*p*-interaction	OR (95% CI)	*p*-interaction	OR (95% CI)	*p*-interaction	OR (95% CI)	*p*-interaction
Age (years) Age categories		**0.785**		**0.369**		**0.292**		**0.518**
60–70	Reference		Reference		Reference		Reference	
70–80	16.13 (0.35, 745.84)		3.13 (0.09, 112.75)		3.48 (0.09, 139.53)		3.27 (0.11, 93.85)	
>80	170.10 (1.70, 17032.00)		14.36 (0.24, 863.12)		5.10 (0.09, 289.37)		179.77 (3.22, 10044.44)	
Gender *n* (%)		**0.338**		**0.298**		**0.702**		**0.649**
Male	Reference		Reference		Reference		Reference	
Female	0.59 (0.03, 13.24)		0.19 (0.01, 3.70)		0.31 (0.02, 5.88)		0.68 (0.05, 9.58)	
Annual family income ($)		**0.688**		**0.001**		**0.943**		**0.513**
<$20,000	Reference		Reference		Reference		Reference	
$20,000to < $45,000	1.18 (0.03, 48.51)		0.06 (0.00, 2.46)		0.31 (0.01, 13.62)		0.26 (0.01, 7.99)	
$45,000to < $75,000	–		0.05 (0.00, 9.31)		0.13 (0.00, 24.26)		0.27 (0.00, 27.37)	
≥$75,000	–		0.10 (0.00, 25.48)		1.79 (0.01, 383.12)		0.11 (0.00, 28.99)	
Alcohol		**0.925**		**0.584**		**0.459**		**0.872**
Yes	Reference		Reference		Reference		Reference	
No	0.10 (0.00, 2.58)		0.32 (0.02, 6.64)		3.60 (0.18, 72.39)		0.63 (0.04, 11.14)	
Body mass weight (kg/m^2^)		**0.125**		**0.992**		**0.964**		**0.253**
Normalweigh (<25)	Reference		Reference		Reference		Reference	
Overweight (25 to <30)	0.06 (0.00, 3.43)		2.39 (0.07, 79.94)		3.21 (0.07, 148.29)		2.17 (0.07, 70.30)	
Obese (> = 30)	0.32 (0.00, 20.40)		1.70 (0.03, 83.30)		12.64 (0.21, 761.40)		5.14 (0.13, 208.44)	
Hypertension		**0.453**		**0.600**		**0.691**		**0.986**
Have hypertension	Reference		Reference		Reference		Reference	
Haven’t hypertension	2.39 (0.08, 69.84)		0.57 (0.02, 14.14)		1.45 (0.06, 32.84)		0.58 (0.03, 10.06)	

The bold black font indicates the *p*-value.

## 4 Discussion

### 4.1 Summary of results

Our analysis of NHANES data from 2011 to 2014, involving 1,544 older adult participants, suggests an association between insecticides exposure and an increased risk of cognitive impairment, particularly in memory and delayed recall as measured by the CERAD-WL and CERAD-DR tests. However, after adjusting for sociodemographic and lifestyle variables, associations were no longer statistically significant. These findings indicate that the relationship between insecticides exposure and cognitive function may be influenced by various confounding factors and warrants further investigation.

### 4.2 Potential biological mechanisms between insecticides exposure and increased cognitive function risk

Exposure to insecticides is suspected to elevate the risk of cognitive dysfunction, yet the mechanisms remain not fully elucidated. Existing research suggests that short-term exposure to insecticides, regardless of the type of insecticide, can have long-term neurobehavioural effects in vertebrates (29) and insects (30) and cause a range of health problems (31). A mouse model of acute organophosphorus poisoning was constructed and showed lipid peroxidation, downregulation of antioxidant enzymes and astrocytosis in the hippocampus and prefrontal cortex, as well as altered dopamine levels, molecular and neurochemical changes that have been associated with long-term memory deficits (32). These compounds are known to inhibit acetylcholinesterase activity, resulting in abnormal accumulation of the neurotransmitter acetylcholine within the nervous system (33). Such accumulation may perturb normal neural transmission, consequently impairing cognitive function (34). In the context of aging, acetylcholine levels in the cerebral cortex diminish, a decline that serves as a critical biomarker for assessing brain health, particularly in conditions of dementia and end-stage (35). Additionally, insecticides are implicated in augmenting free radical production, which can induce oxidative stress damaging cell membranes and DNA, thereby compromising neuronal health (36). Studies have found that chronic, low-dose exposure to certain insecticides can inhibit mitochondrial complex I and cytochrome oxidase in the brain. This inhibition increases the production of reactive oxygen species, which can disrupt the cell’s antioxidant defense system. As a consequence, the release of cytochrome c from the mitochondria into the cytoplasm leads to apoptosis (37). There is also evidence that insecticides may activate microglia, initiating neuroinflammation, a process associated with the development of neurodegenerative diseases (38). Animal experiments have demonstrated that even low doses of certain insecticides can induce a pro-inflammatory state in the brain during subchronic exposure stages, and can enhance the neuroinflammatory response to lipopolysaccharide in a region-specific manner (39). These findings provide important biological insights into the mechanisms by which insecticides impact cognitive function and suggest potential directions for future research.

### 4.3 Analysis of subgroup results

Subgroup analysis revealed a striking phenomenon: exposure to insecticides significantly impacted the cognitive function of men aged 70 to 80 and individuals with an annual income between $20,000 and $45,000. This finding may be related to the natural increase in the risk of cognitive disorders in the 70 to 80 age group, especially in regions and populations more likely to be involved in the mixing and application of pesticides, thereby increasing exposure risk. But studies have confirmed that environmental toxins, including pesticides, have been identified as important contributors to cardiovascular and brain aging diseases. These toxic substances cause damage to the large blood vessels and microvessels, and many of them also cross the blood–brain barrier, inducing neurotoxic effects, neuroinflammation and neuronal dysfunction. In conclusion, environmental factors play a crucial role in regulating cardiovascular and cerebral aging (40). There was also a study comparing farmers exposed to pesticides with a control group not exposed to pesticides, and significant changes were found in the farmers’ group: increased oxidative DNA damage, decreased acetylcholinesterase activity, and elevated levels of IL-10 and CRP (41). And it has been previously demonstrated that short-term exposure to organophosphorus pesticides results in cognitive damage (42) and central system disorders (43) in mice. Furthermore, individuals with an annual income between $20,000 and $45,000 may face more severe external environmental conditions, which could imply a higher likelihood of exposure to pesticides. Evidence shows that the risk of cognitive dysfunction is exacerbated in environments with high levels of insecticides exposure (44). Insecticides may damage cognitive function through mechanisms such as inducing oxidative stress, inflammatory responses, cholinergic neurotoxicity, and mitochondrial dysfunction (45). As age increases, these factors contribute to a higher incidence of cognitive disorders, particularly among men over 60, making it a significant health issue.

### 4.4 Analysis of specific urinary metabolites

To further explore the relationship between insecticide exposure and cognitive function, we analysed three specific urinary metabolites: trans-DCCA, PNP and 2,4-D. These metabolites correspond toorganophosphorus insecticides, pyrethroid insecticides and a herbicide, respectively. Our findings revealed differential associations between metabolite concentrations and cognitive function indices. Notably, the results of trans-DCCA demonstrated a statistically significant association with CERAD-WL scores (adjusted OR: 0.94, 95% CI: 0.90–0.99, *p* = 0.018), which further confirms the existence of a potential effect of insecticide exposure on cognitive function. The findings align with previous research, It has been shown that prenatal exposure to higher concentrations of trans-DCCA, a metabolite of pyrethroid insecticides, was significantly associated with a 2.24-point decrease in Mental Development Index (MDI) scores and a 1.90-point decrease in Psychomotor Development Index (PDI) scores in boys for every unit increase in first-trimester urine, suggesting that Trans-DCCA has a negative impact on children’s cognitive and neurological development (46). Furthermore, prenatal exposure to pyrethroid insecticides may contribute to neurodevelopmental issues in children, particularly when maternal urine concentrations of trans-DCCA exceed detectable thresholds. Consequently, children are at an increased risk of developing symptoms consistent with ADHD, underscoring the potentially detrimental neurodevelopmental effects of trans-DCCA (47). These findings underscore the harmful implications of Trans-DCCA on neurological function. However, other indicators and metabolites in this study did not show statistically significant associations, which highlights the complexity of the relationship between specific insecticide metabolites and cognitive function, and requires more in-depth investigation in future studies.

### 4.5 Potential association between insecticides exposure and cognitive impairment: limitations in establishing dose–response relationships

Our study suggests a potential association between exposure to insecticides and cognitive impairment, aligning with existing evidence on pesticide neurotoxicity. The absence of exposure gradient data precludes a definitive dose–response assessment, posing a critical limitation for causal inference. Nevertheless, existing literature and biological plausibility provide a basis for suggesting that the dose–response relationship is a promising area for further investigation. For instance, a recent study suggests a significant association between pesticide exposure and cognitive impairment. Chronic exposure to chemical mixtures, including pesticides, is strongly associated with oxidative stress, inflammation and neurodegenerative diseases. Regardless of the dose, pesticide exposure may adversely affect cognitive function through mechanisms such as neuroinflammation and immune activation (48). In Akwesasne Mohawk older adults, high concentrations of PCBs, HCB and DDE were significantly associated with greater cognitive decline. In contrast, among older adults in NHANES, cognitive decline was primarily associated with exposure to highly chlorinated PCBs and DDE. Significant differences in the effects of various chemicals mixtures on cognitive function were observed, with higher the exposure doses correlating with a greater the risk of cognitive decline (49). It has also been shown that there is a significant U-shaped relationship between the exposure dose of 2,4-dichlorophenoxyacetic acid (2,4-D) and cognitive function in the older adult. Generalized linear models showed that high-dose exposure may negatively affect cognitive function. Furthermore, restricted cubic spline regression and generalized additive modelling further revealed a U-shaped relationship between 2,4-D exposure and cognitive function, suggesting that low and high-dose exposures may have different effects on cognitive function. This study highlights that appropriate control of the range of 2,4-D exposure is particularly important for cognitive function, especially among men (50). Because this paper examines chronic low-dose exposures in the home environment, there are a study in Spain showed a significant association between persistent organic pollutants (POPs) and cognitive decline in Hispanic/Latino adults. The study found that each doubling of plasma levels of polychlorinated biphenyls (PCBs) 146, 178, 194, 199/206, and 209 were associated with steeper global cognitive decline (51). There are also some studies suggesting that chronic low-dose exposure to these insecticides may result in neurobehavioural changes and alterations at the molecular level, suggesting potential neurotoxic risks to aquatic organisms (52) and humans (53). Although there is room for improvement in the detailed portrayal of the exposure gradient data in this study, the results of previous studies provide a solid foundation to support the potential link between exposure to insecticides and cognitive impairment observed in our study.

### 4.6 Strengths and limitations

This study has several important strengths: firstly, we employed three models: weighted multivariate logistic regression, study population description, and subgroup analysis, to comprehensively explore the complexity of how insecticides exposure affects cognitive function. Secondly, the use of the NHANES database, with its large sample size, rich scientific variables, and coverage of multi-stage complex studies, provided valuable data resources for this research (54). Additionally, this study included a variety of confounding variables based on existing research, significantly reducing potential biases from these factors.

However, this study also has some admitted limitations. Despite NHANES being an authoritative data source, it does not include all relevant variables that may affect the relationship between pesticide use and cognitive function. Therefore, while we adjusted for a range of sociodemographic and lifestyle variables, the possibility of residual confounding remains. Unmeasured confounders, such as occupational exposure, rurality, genetic predisposition, and detailed environmental factors, were not accounted for in our analysis. These factors could independently influence cognitive function and, if not adequately controlled, may lead to biased estimates of the association between insecticides exposure and cognitive impairment.

Furthermore, exposure assessment in our study relied on self-reported data regarding pesticide use in domestic, horticultural, and garden contexts. Self-reported data are prone to recall bias and may not accurately reflect actual exposure levels. For instance, participants may overestimate or underestimate their exposure, leading to non-differential misclassification. This type of misclassification tends to bias effect estimates toward the null, potentially masking true associations. Also, insecticides exposures are assessed primarily on the basis of a 7-day recall period, which may not adequately reflect cumulative or chronic exposures. This shorter recall period may be subject to recall bias, as participants may not accurately remember their exposure over a longer period of time.

In addition to this, our study conducted multiple statistical tests to evaluate the association between insecticides exposure and various cognitive outcomes. This approach increases the risk of alpha inflation, where the probability of obtaining a statistically significant result by chance alone (Type I error) is higher than the nominal alpha level (typically 0.05). Given the number of tests performed, some of our significant findings may be due to chance rather than true associations. Future studies should aim to assess cumulative insecticides exposure over an extended period to better capture the chronic nature of exposure.

## 5 Conclusion

Our findings suggest an association between insecticides exposure and an increased risk of cognitive impairment, particularly in memory and delayed recall as measured by the CERAD-WL and CERAD-DR tests. However, the confidence intervals for some associations were close to the null value, indicating weak evidence for a causal relationship. And it is the cross-sectional study design, we cannot infer causality from these findings. In addition, we need to consider the possibility of reverse causation, particularly due to the cross-sectional nature of the study. Reverse causation means that cognitive impairment may affect reporting or recall of pesticide exposure, rather than exposure causing cognitive impairment. For example, individuals with cognitive impairment may have a reduced ability to accurately recall or identify their pesticide exposure. This may lead to underreporting or overreporting of exposure, introducing bias into the analysis.

So future studies should employ longitudinal designs to assess the long-term effects of insecticides exposure on cognitive function. The use of biological markers, such as urinary metabolites of insecticides, can provide a more accurate exposure assessment. Additionally, advanced statistical methods, such as mediation analysis and structural equation modelling, should be applied to disentangle the complex relationships between exposure, confounders, and outcomes. Collecting detailed data on potential confounders, such as genetic predisposition, socioeconomic status, and lifestyle factors, will enhance the robustness of causal inferences.

In addition, we acknowledge that a limitation of our study is the exclusion of individuals below the age of 60. Early-onset dementia can develop in individuals over the age of 40, and including a younger age group could potentially reveal associations between pesticide exposure and the onset of dementia. Future research would benefit from including a broader range of ages to comprehensively evaluate the impact of pesticides on cognitive function across different age groups. Emphasizing early detection is crucial for effective disease management. Additionally, exploring the relationship between pesticide exposure and early-onset dementia could provide valuable insights for developing better prevention and intervention strategies.

In conclusion, while our study provides preliminary evidence of an association between insecticides exposure and cognitive impairment, further research is needed to establish causality and inform preventive strategies.

## Data Availability

The datasets presented in this study can be found in online repositories. The names of the repository/repositories and accession number(s) can be found at: https://www.cdc.gov/nchs/nhanes/.

## Footnotes

^1^ http://www.cdc.gov/nchs/nhanes/
