# Supplementary material for: Association between insecticide exposomics and cognitive function in older adults: an observational study based on NHANES 2011–2014
Source: Front Public Health. 2025 Jul 9;13:1556263. doi: 10.3389/fpubh.2025.1556263 (PMC12283784; doi:10.3389/fpubh.2025.1556263)
Supplement: Supplementary file 1 [file Data_Sheet_1.docx]

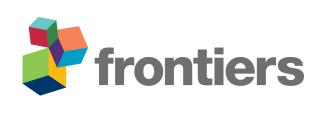
 ***Supplementary Material***

| **Trans-DCCA** |  | 0 (N=101) | 1 (N=491) | OR (univariable) |
| --- | --- | --- | --- | --- |
| **CERAD-DR** | Mean ± SD | 6.2 ± 2.1 | 5.7 ± 2.2 | 0.91 (0.83-1.01) *p=0.072* |
| **DSST** | Mean ± SD | 43.8 ± 17.0 | 43.8 ± 17.3 | 1.00 (0.99-1.01) *p=0.998* |
| **AFT** | Mean ± SD | 16.5 ± 5.0 | 16.2 ± 5.3 | 0.99 (0.95-1.03) *p=0.570* |
| **CERAD-WL** | Mean ± SD | 19.6 ± 4.0 | 18.4 ± 4.7 | 0.94 (0.90-0.99) *p=0.018* |
| \| **PNP** \| \|  \| 0 (N=574) \| 1 (N=18) \| OR (univariable) \| \| --- \| --- \| --- \| --- \| --- \| --- \| \| **CERAD-DR** \| Mean ± SD \| \| 5.8 ± 2.2 \| 5.1 ± 2.5 \| 0.87 (0.71-1.07) *p=0.188* \| \| **DSST** \| Mean ± SD \| \| 43.7 ± 17.2 \| 48.2 ± 17.5 \| 1.02 (0.99-1.04) *p=0.270* \| \| **AFT** \| Mean ± SD \| \| 16.2 ± 5.3 \| 16.6 ± 4.7 \| 1.01 (0.93-1.10) *p=0.786* \| \| \| **2,4-D** \|  \| 0 (N=451) \| 1 (N=141) \| OR (univariable) \| \| --- \| --- \| --- \| --- \| --- \| \| **CERAD-DR** \| Mean ± SD \| 5.8 ± 2.3 \| 5.8 ± 2.1 \| 1.01 (0.93-1.10) *p=0.827* \| \| **DSST** \| Mean ± SD \| 44.0 ± 17.4 \| 43.2 ± 16.6 \| 1.00 (0.99-1.01) *p=0.626* \| \| **AFT** \| Mean ± SD \| 16.4 ± 5.4 \| 15.7 ± 5.0 \| 0.97 (0.94-1.01) *p=0.143* \| \| **CERAD-WL** \| Mean ± SD \| 18.7 ± 4.8 \| 18.4 ± 3.9 \| 0.99 (0.95-1.03) *p=0.516* \| \| \| \| \| \| \| | | | | |

**Supplementary Table 1:** Associations between insecticides metabolites levels and Increased Cognitive Function Risk

The data on the graph represents a multivariate logistic regression between 3 urinary metabolites of insecticides and cognitive function from 2011-2014, with 592 participants remaining after data screening. The metabolites shown are: Trans-3-(2,2-dichlorovinyl)-2,2-dimethylcyclopropane carboxylic acid (Trans-DCCA) (organophosphate insecticides) ; para-nitrophenol (PNP) (pyrethroids), and a herbicide—2,4-dichlorophenoxyacetic acid (2,4-D) .

The value “0” means that the result was at or above the limit of detection, “1” indicates that the result was below the limit of detection.

**Supplementary Table 2:** The lower limit of detection (LLOD, in ug/L) for Trans-DCCA, 2,4-D, & PNP:

| Acronyms | Analyte Description | LLOD |
| --- | --- | --- |
| Trans-DCCA | trans-dichlorovinyl dimethylcyclopropane carboxylic acid | 0.60 |
| PNP | *para*-Nitrophenol | 0.10 |
| 2,4-D | 2,4-dicholorphenoxyacetic acid | 0.15 |

**Supplementary Table 3:** Multivariate logistic regression between OPPs exposure and increased cognitive function risk in the National Health and Nutrition Examination Survey conducted in the US between 2011-2014.

| **Defend insect** |  | yes(N=191) | no(N=1200) | reject(N=6) | OR (univariable) |
| --- | --- | --- | --- | --- | --- |
| **CERAD-WL(<17)** | Mean ± SD | 19.2 ± 4.6 | 19.8 ± 4.6 | 17.3 ± 5.5 | 1.03 (0.99-1.06, *p=0.102*) |
| **CERAD-DR(<5)** | Mean ± SD | 5.9 ± 2.3 | 6.3 ± 2.3 | 5.3 ± 2.9 | 1.08 (1.02-1.16, *p=0.014*) |
| **AFT(<14)** | Mean ± SD | 16.6 ± 5.8 | 16.9 ± 5.4 | 13.3 ± 5.0 | 1.01 (0.98-1.04, *p=0.499*) |
| **DSST(<34)** | Mean ± SD | 42.7 ± 18.1 | 47.5 ± 16.6 | 31.0 ± 13.5 | 1.02 (1.01-1.03, *p<0.001*) |

| **Kill weeds** |  | yes (N=142) | no (N=1221) | reject(N=3) | OR (univariable) |
| --- | --- | --- | --- | --- | --- |
| **CERAD-WL(<17)** | Mean ± SD | 19.7 ± 4.6 | 19.7 ± 4.6 | 20.3 ± 5.2 | 1.00 (0.97-1.04, *p=0.916*) |
| **CERAD-DR(<5)** | Mean ± SD | 6.2 ± 2.3 | 6.3 ± 2.3 | 6.4 ± 3.0 | 1.02 (0.94-1.10, *p=0.672*) |
| **AFT(<14)** | Mean ± SD | 17.6 ± 5.3 | 16.7 ± 5.4 | 18.3 ± 6.5 | 0.97 (0.94-1.00, *p=0.065*) |
| **DSST(<34)** | Mean ± SD | 49.0 ± 17.5 | 46.4 ± 16.7 | 50.9 ± 18.5 | 0.99 (0.98-1.00, *p=0.093*) |

A total of 146 cases of missing data were removed in this study, and the total number of cases analysed was 1398.

**Supplementary Table 4: STROBE Checklist**: Items for Reporting in Observational Studies in Epidemiology

| Item No. | Item Description | Compliance | Notes |
| --- | --- | --- | --- |
| 1 | Title | Yes | The title explicitly indicates that the study is observational. |
| 2 | Abstract | Yes | The abstract includes a brief background, objectives, methods, results, and conclusions. |
| 3 | Introduction | Yes | The study background and objectives are detailed. |
| 4 | Methods | Yes | The study design, data sources, sample selection, and statistical methods are described. |
| 5 | Results | Yes | Main and secondary results are reported, including effect estimates and confidence intervals. |
| 6 | Discussion | Yes | The significance of the results, limitations, and recommendations for future research are discussed. |
| 7 | Conclusion | Yes | The conclusion summarizes the key findings and emphasizes the observational nature of the study. |
| 8 | Tables and Figures | Yes | All tables and figures include complete variable definitions, units, and reference categories. |
| 9 | Multiple Comparisons | Yes | The issue of multiple comparisons is discussed, and correction methods are proposed. |
| 10 | Sensitivity Analysis | Yes | Sensitivity analysis was conducted, and the results are reported. |
| 11 | Residual Confounding and Exposure Misclassification | Yes | The potential impact of residual confounding and exposure misclassification is discussed. |
| 12 | Reverse Causality | Yes | The possibility of reverse causality is discussed, and strategies to address it are proposed. |
| 13 | Dose-Response Relationship | Yes | The dose-response relationship is discussed, and recommendations for future research are made. |
| 14 | Bradford-Hill Criteria for Causality | Yes | The Bradford-Hill criteria are applied to assess causality. |

**STROBE Checklist Confirmation**

We confirm that this paper complies with all items of the STROBE Checklist. Each item has been fully addressed in the text, ensuring the transparency and scientific rigor of the study.
